# Recurrent *CAPN3* p.Asp753Asn Variant Supports a Potential Dominant Calpainopathy with Variable Clinical Expressivity

**DOI:** 10.3390/ijms262311384

**Published:** 2025-11-25

**Authors:** Giorgia D’Este, Alejandro Giorgetti, Denise Cassandrini, Francesca Magri, Dario Ronchi, Anna Rubegni, Diego Lopergolo, Alessandro Malandrini, Luciano Merlini, Gaetano Vattemi, Paola Tonin, Rita Barresi

**Affiliations:** 1 Neurobiology Laboratory, San Camillo IRCCS, Via Alberoni 70, 30126 Venezia, Italy; 2 Department of Biotechnology, University of Verona, Strada Le Grazie 15, 37134 Verona, Italy; 3 UOC Immunologia, Azienda Ospedaliera Universitaria Integrata, P.le L.A. Scuro 10, 37134 Verona, Italy; 4 Department of Neurosciences, Biomedicine and Movement Sciences, Section of Clinical Neurology, University of Verona, Piazzale L.A. Scuro 10, 37134 Verona, Italy; 5 Neuromuscular Unit, IRCCS Fondazione Ca’ Granda Ospedale Maggiore Policlinico, Via Francesco Sforza 28, 20122 Milan, Italy; 6 Neurology Unit, IRCCS Fondazione Ca’ Granda Ospedale Maggiore Policlinico, 20122 Milan, Italy; 7 Dino Ferrari Center, University of Milan, Via Francesco Sforza 35, 20122 Milan, Italy; 8 IRCCS Stella Maris Foundation, Viale del Tirreno 341, 56128 Pisa, Italy; 9 Department of Medicine, Surgery and Neurosciences, University of Siena, 53100 Siena, Italy; 10 UOC Neurologia, Azienda Ospedaliero-Universitaria Senese, Viale Bracci 16, 53100 Siena, Italy; 11 Department of Biomedical and Neuromotor Science (DIBINEM), University of Bologna, Piazza di Porta S. Donato 2, 40127 Bologna, Italy

**Keywords:** limb-girdle muscular dystrophy, calpainopathy, *CAPN3*, dominant inheritance

## Abstract

Limb-Girdle Muscular Dystrophies (LGMDs) are genetically heterogeneous disorders primarily affecting proximal limb muscles. The most common form, LGMDR1, results from biallelic *CAPN3* mutations encoding calpain-3, a muscle-specific protease. Recently, growing evidence implicates heterozygous *CAPN3* variants in autosomal dominant disease (LGMDD4), with pathogenic mechanisms still incompletely understood. In a retrospective multicenter Italian study of patients harboring monoallelic *CAPN3* variants (ClinicalTrials.gov NCT05956132), the p.Asp753Asn substitution was the most frequent change, detected in eight unrelated individuals. These patients, aged 6–80 years, exhibited a spectrum of presentations ranging from asymptomatic hyperCKemia and exertional myalgia to mild proximal weakness. Muscle biopsies showed mild, nonspecific myopathic changes, while calpain-3 expression was variably reduced. Structural modeling suggested that Asp753 may stabilize the Ca^2+^-bound conformation, with substitution potentially disrupting inter-domain interactions. Literature review identified 31 additional reports worldwide, confirming recurrence while highlighting marked phenotypic heterogeneity and limited clinical annotation. The aggregated evidence supports a pathogenic role for p.Asp753Asn, though the precise mechanism, potentially involving a dominant-negative effect, remains to be validated. These findings emphasize diagnostic challenges posed by single *CAPN3* variants and underscore the need for integrated clinical, segregation, and functional studies to clarify pathogenic mechanisms, refine counseling, and guide patient-specific rehabilitation and therapeutic strategies.

## 1. Introduction

Limb-Girdle Muscular Dystrophies (LGMDs) comprise a genetically and clinically diverse group of disorders primarily affecting the muscles of the pelvic and shoulder girdles. The most prevalent form, LGMDR1 (formerly LGMD2A), arises from biallelic loss-of-function mutations in *CAPN3* (MIM# 114240), encoding calpain-3, an intracellular cysteine protease primarily expressed in skeletal muscle [1,2,3]. Calpain-3 is characterized by a modular structure with distinctive functional domains, which includes protease core domains PC1 and PC2, the calpain-type beta-sandwich (CBSW) domain and a C-terminal penta-EF-hand (PEF) domain which forms a stable functional dimer upon Ca^2+^ binding [4,5]. It also harbors sequences designated for autolytic and proteolytic activity and interaction with structural and regulatory muscle proteins, such as titin and filamin C [6,7]. Physiologically, calpain-3 plays a critical role in maintaining muscle homeostasis through sarcomere remodeling, proteostasis, and calcium signaling, processes essential for muscle repair and regeneration [8]. Current studies indicate that calpain-3 operates as an active homodimer, although it may also exist in different oligomeric states possibly modulated by cellular context or post-translational mechanisms, which in turn may affect its stability and function [9,10]. To date, over 500 mutations in *CAPN3* have been identified, the majority of which are missense mutations [11].

The pathogenesis of LGMDR1 involves a loss of calpain-3 expression and/or function. Importantly, loss-of-function mutations in *CAPN3* do not always result in complete absence of calpain-3 protein, and expression levels do not consistently correlate with clinical severity, highlighting that pathogenicity is not solely dependent on protein abundance. Except in cases of complete absence or severely reduced calpain-3, Western blot (WB) is not a clear diagnostic indicator. In healthy muscle, a distinct pattern of bands is consistently observed by WB: the full-length protein at 94 kDa, a 30 kDa autolytic fragment, and ~60 kDa degradation products. In LGMDR1 patients, these banding patterns can vary significantly. Severely affected individuals often show loss of all bands, typically linked to null or truncating mutations [12], while others may retain the 94 kDa band but lack the 30 and ~60 kDa fragments, indicating enzymatically inactive protein due to defective autolysis [13]. Therefore, the presence of calpain-3 protein does not necessarily indicate functional integrity, and accurate interpretation should integrate information from the analysis of all calpain-3 bands as a whole [14].

While calpainopathies are classically inherited in an autosomal recessive manner, a small number of cases with autosomal dominant inheritance (LGMDD4) have also been reported and linked to specific *CAPN3* mutations [15,16,17,18,19,20,21,22]. Clinically, LGMDD4 is often milder and later in onset than LGMDR1, with patients typically presenting in the third or fourth decade with limb-girdle weakness, often accompanied by myalgia and axial symptoms [15]. Notably, some of the mutations reported in compound heterozygous or homozygous form in patients with LGMDR1, have also been found in heterozygosity in individuals with dominantly inherited disease [15,19,22]. In such cases, segregation analyses within affected families have demonstrated consistent transmission of muscle weakness with the heterozygous variant, confirming a dominant inheritance pattern. Research on the pathogenic mechanism of LGMDD4 has largely focused on loss-of-function mechanisms employing techniques such as co-transfection analyses or in silico simulations [19,20]. One proposed model for dominant-negative pathogenicity hypothesizes that mutant monomers combine with wild-type monomers, leading to faulty dimers [15] which may be inactive or rapidly degraded, resulting in a level of wild-type calpain-3 insufficient to maintain muscle homeostasis. Interestingly, despite this hypothesis, protein expression in muscle biopsies from affected individuals often appears normal [18,23] or even lower than expected, assuming that wild-type and mutant monomers combine in a simple stoichiometric ratio [15,19]. Such mechanism remains speculative due to the current lack of reliable methods and biomarkers for confirming dominant pathogenicity in calpainopathies. The complexity of protein interactions, oligomerization states, and tissue-specific effects complicates the ability to assess the biological effect of single missense variants. Indeed, while nonsense, insertion/deletion, or splice-site mutations are likely to impair or eliminate calpain-3 function, the impact of missense or in-frame mutations on calpain-3 activity is not immediately predictable [24]. Many additional variants, beyond those described, may contribute individually to the development of LGMDD4. Indeed, a dominant form of calpainopathy may constitute a possible diagnosis in the relatively high proportion (~20%) of LGMD cases where only a single variant in *CAPN3* is identified [25]. In sporadic cases, multiple reports of individuals with the same heterozygous *CAPN3* variant and presenting with a compatible phenotype may provide circumstantial but compelling evidence for a dominant pathogenic effect. In this context, the missense variant c.2257G>A (p.Asp753Asn) emerged as a recurrent finding in our multicenter cohort of unrelated Italian patients with monoallelic changes in *CAPN3*. This variant has been extensively reported in both heterozygous and compound heterozygous states in literature [17,25,26,27,28,29,30,31,32,33,34,35,36] and is currently classified as uncertain significance in the ClinVar database. In the present study, we aim to further investigate the potential role of the p.Asp753Asn substitution in the pathogenesis of LGMDD4 by consolidating existing literature and analyzing clinical and biochemical data from our cohort.

## 2. Results

### 2.1. Subjects

In a retrospective, observational, multicenter study (Ethics Committee Approval 2023.08; 6 June 2023, ClinicalTrials.gov NCT05956132), we gathered a cohort of 59 Italian patients heterozygous for *CAPN3* variants. A comprehensive analysis of the entire dataset is beyond the scope of the present report and will be presented in a separate publication. Genetic analysis identified 39 distinct variants in total. The most frequent variant was c.2257G>A (p.Asp753Asn), located in exon 21, accounting for 13.5% of cases. This subset of patients represents the focus of the present study.

### 2.2. Clinical, Histopathological and Biochemical Features of p.Asp753Asn Patients

Demographic and clinical characteristics of the patients are summarized in Table 1.

The study cohort consists of eight unrelated individuals, six males and two females, including one pediatric subject (6 years of age) and 7 adult cases with an age at last evaluation ranging from 52 to 80 years (mean age 63 ± 10 years). Age of onset in adult cases ranged between the fourth and the sixth decade of life (mean age 50.1 ± 7.1 years). All were found to be heterozygous for the p.Asp753Asn variant, with no additional variants identified in *CAPN3*. Although one patient (P8) carried a variant in another gene associated with muscular dystrophy (*POMGNT2*), it was considered unlikely to be causative. Family history was negative for all cases except P8, whose brother presented with pes cavus and hyperCKemia and whose daughter had hyperCKemia without clinical symptoms. This is suggestive of a possible dominant inheritance pattern, although segregation analysis could not be confirmed due to the absence of genetic testing in the affected relatives.

The clinical spectrum was very broad. Overall, five patients experienced muscular atrophy and weakness (P2, P3, P4, P7, P8), which was mainly distributed in proximal muscles of lower limbs determining progressive difficulty in walking and/or climbing stairs, frequent falls, or foot drop as initial symptoms. Interestingly two patients (P3 and P7) showed also a severe axial weakness with bent spine syndrome and one patient (P4) presented with a distal predominant involvement. One patient carried asymptomatic hyperCKemia at 56 years of age with normal neurological examination (P1). Three patients (P5, P6, P8) reported myalgia, exercise intolerance, or fatigue. Contractures and respiratory symptoms were present in one patient (P7) while cardiac involvement was not described in the entire cohort.

Creatine kinase (CK) levels ranged from normal to mild increased values (up to 5× normal, range normal-1100 U/L). Interestingly, no correlation was observed between muscle impairment and serum CK levels, as patients with muscular weakness could also present with normal CK values. Muscle MRI or CT scan was performed in 6/8 patients showing unremarkable pattern in 3 of them (P1, asymptomatic, and P4 and P5, showing weakness) and fibro-fatty replacement in 3 subjects (P3, P7, P8).

**Figure 1:**
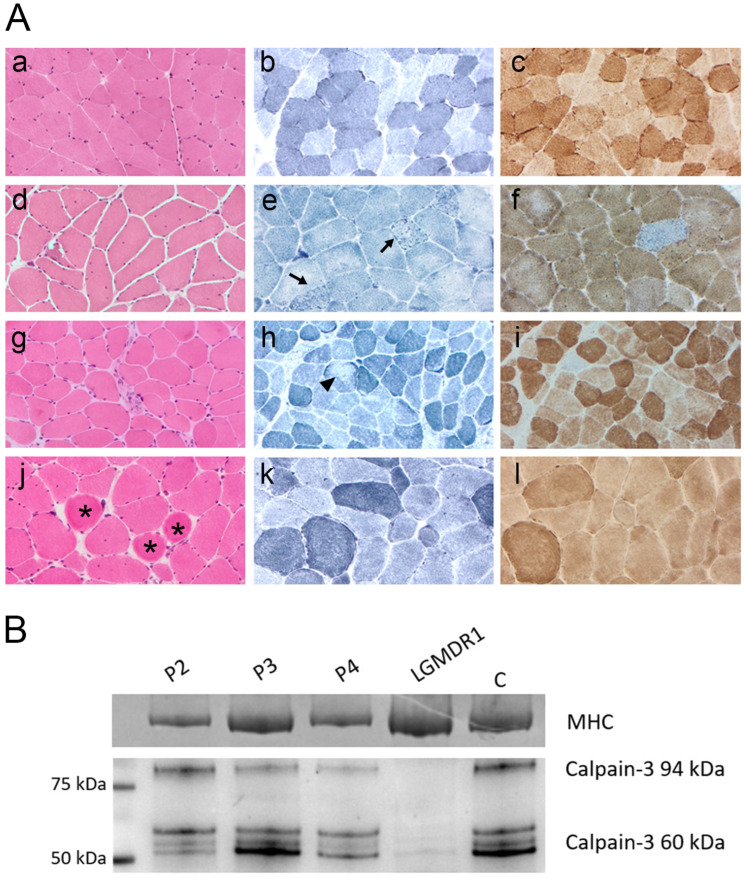
(**A**) Muscle biopsy analysis. Representative morphological findings in patients P2, P3, and P4 compared with healthy control muscle (**a**–**c**). Panels (**d**–**f**), (**g**–**i**), and (**j**–**l**) correspond to patients P2, P3, and P4, respectively. H&E staining (**d**,**g**,**j**) showed marked fiber size variability, internal nuclei, rimmed vacuoles, endomysial fibrosis, and ring fibers (asterisks). NADH staining (**e**,**h**,**k**) revealed focal and irregular areas of reduced oxidative activity (arrows and arrowhead). COX staining identified occasional COX-negative fibers. Magnification: ×20. (**B**) Western blot analysis. Muscle biopsy samples from patients P2–P4 were analyzed by WB to assess calpain-3 expression. The blot shows the full-length 94 kDa calpain-3 protein and its autolytic/degradation fragments (~60 kDa) in comparison with a healthy control (C). Patient P2 showed calpain-3 expression within normal range, P3 exhibited a reduction in the 94 kDa band, and P4 displayed an overall reduction in all calpain-3 bands. A muscle biopsy from a patient diagnosed with LGMDR1 showing absence of the 94 kDa band and severe reduction in the autolytic/degradation fragments was included as a negative control. Total protein loading was confirmed by Coomassie blue staining of the myosin heavy chain (MHC) in the post-blotted gel.

Muscle biopsy was performed in 6/8 patients and showed mild myopathic features in all of them with mild fiber size variability and few internal nuclei. Endomysial fibrosis was described in 3/6 samples (P1, P2, P3). Inflammatory infiltrates were absent. Degenerating muscle fibers, rimmed vacuoles, ring fibers, fibers with loss or reduction in oxidative enzyme activity and COX negative fibers were also observed occasionally (Figure 1A). Some nonspecific signs such as increase in PAS-positive material within the intermyofibrillar network, predominance of type I fibers, lobulated muscle fibers were described only in some samples but did not represent a key feature. WB analysis of calpain-3 performed in five specimens showed a normal expression of the protein in three patients (e.g., P2, Figure 1B) while in the other two calpain-3 expression was variably reduced (e.g., P3 and P4, Figure 1B).

### 2.3. Structural Modeling of the p.Asp753Asn Variant

**Figure 2:**
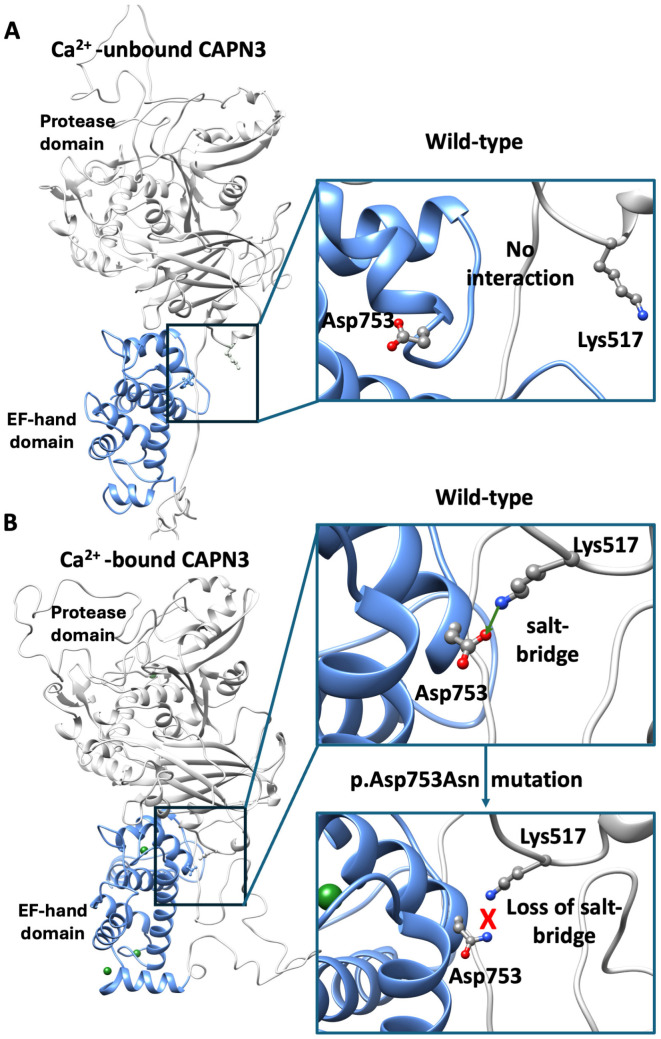
Structural modeling of full-length calpain-3. (**A**) Homology model of full-length calpain-3 in the Ca^2+^-unbound state, Asp753 within the penta EF-domain (light blue) and Lys517 within the protease domain (gray) are highlighted. In the insight, the detailed location of both residues is shown. (**B**) Ca^2+^-bound model showing the formation of the Asp753–Lys517 salt bridge, consistent with Ca^2+^-induced conformational rearrangements. In the lower close-up view, the mutant model (p.Asp753Asn) illustrates the loss of the negative charge and the predicted breaking of the salt-bridge.

In order to investigate the potential effect of the p.Asp753Asn mutation on calpain-3 structure and function, it is essential to rely on structural information about the protein. However, no full-length experimental structure of calpain-3 is currently available. Instead, structural data exist for the penta-EF-hand domain (PDB: 4OKH) and for several structures of the protease core domain (PDB: 6BJD, 6BDT, 6BKJ, 6BJP). Thus, for obtaining insights into the full-length protein in the Ca^2+^-bound and unbound states, homology modelling was performed. The templates chosen for both states were the M-calpain in Ca^2+^ bound and unbound states (PDB 3BOW and 1FKU), sharing a sequence identity of 52% with calpain 3. Then the mutation was mapped onto the modeled structures (Figure 2).

As a quality control step, our modeled structures were compared with the available per domain X-ray structures. For the Ca^2+^-bound state, the penta-EF-hand model showed an RMSD of 1 Å from 4OKH, while the protease core model displayed an RMSD of 0.7 Å from 6BGP, indicating high per-domain accuracy. The use of multiple templates allowed us to capture information about domain organization that is otherwise unavailable from isolated domain structures.

**Figure A1:**
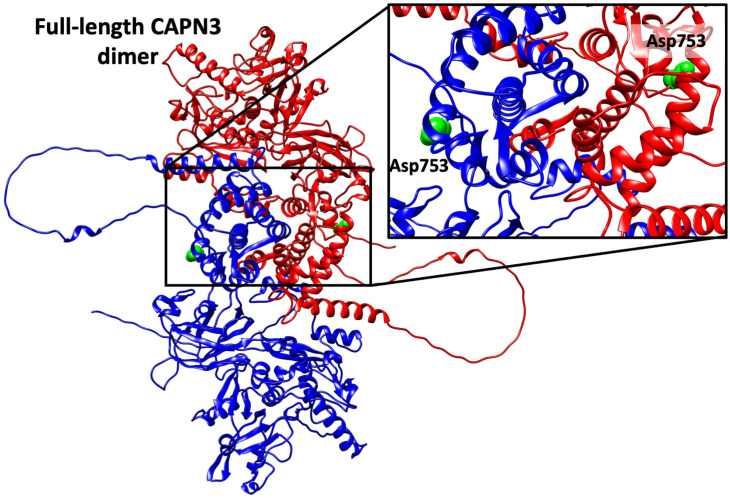
Full-length model of calpain-3. The Alphafold model of the dimer (chain A in blue, and chain B in red) shows that Asp753 residue (green) locates in a region distant to the dimerization interface. Given the limitations of Alphafold in the modeling of domain organization, the model was checked against the crystallographic dimers (4OKH) and the location of Asp753 outside the dimerization interface was confirmed.

Mapping of Asp753 onto the model placed the residue within the PEF-hand domain, but outside of known Ca^2+^-binding sites, calmodulin-binding regions, or multimerization interfaces (Figure A1). Interestingly, in the Ca^2+^-bound model, Asp753 forms a salt bridge with Lys517 of the protease domain, a contact that is disrupted in the Ca^2+^-unbound state (Figure 2). This observation agrees with previous findings by Hanna et al. [37], who reported that Ca^2+^ binding induces conformational rearrangements of PEF domains, driving broader domain reorganization (Supplementary Movie S1). This suggests that Asp753 may contribute to the stabilization of the Ca^2+^-bound conformation and its connection to the protease domain. Substitution of Asp753 with asparagine (p.Asp753Asn) likely removes a negative charge, potentially breaking the salt bridge with Lys517 and destabilizing inter-domain interactions.

### 2.4. The p.Asp753Asn Variant in CAPN3 Is Recurrent Across Unrelated Individuals Worldwide

To contextualize our findings and assess the broader significance of the p.Asp753Asn variant, we reviewed all available literature reports describing patients carrying this substitution. We identified 31 reports of the *CAPN3* variant across 16 independent studies (Table A1). A substantial subset of these reports originated from large-scale sequencing initiatives, including targeted neuromuscular gene panels and whole-exome sequencing (WES) studies of undiagnosed disease cohorts from multiple countries [17,30,31,33]. Consequently, complete patient identifiers and detailed clinical annotations were often unavailable, making it difficult to distinguish between unique individuals, duplicate entries, or related family members. Demographic and phenotypic data were available for only 15 patients (~48%) with key information such as age at onset, family history, zygosity status, and detailed clinical features variably reported or absent. This paucity of clinical annotations significantly limits any robust genotype-phenotype correlation for the p.Asp753Asn substitution.

Genotyping data revealed that the p.Asp753Asn variant most frequently occurred in isolation, with no additional pathogenic *CAPN3* mutations identified in the majority of cases (n = 14). However, compound heterozygous cases were also reported. For example, one individual was a compound heterozygous for p.Asp753Asn and the nonsense mutation c.967G>T (p.Glu323*) [38]. In two cases, two additional *CAPN3* mutations were found in the same patient. Rubegni et al. reported a patient harboring c.1395-1397del (p.Leu465_Glu466del) and c.1453A>G (p.Met485Val) in cis, while p.Asp753Asn was present on the opposite allele [30]; in another report, c.1401_1403delGGA (p.Glu467del) was found on the same allele as p.Asp753Asn in a patient who carried c.967G>T (p.Glu323*) in trans [39]. Two individuals carried intronic variants or rare genomic variants whose clinical significance remains uncertain [34,40], and in two cases, causative variants were detected in other genes, suggesting an alternative molecular diagnosis [35].

Where information was available, most individuals appeared to be of sporadic origin, with no reported family history of neuromuscular disease. Clinical presentation was heterogeneous: among the individuals with reported phenotypic features, varied widely from pediatric (1–3 years) to adult (21–55 years). Phenotypes ranged from asymptomatic hyperCKemia (n = 2) to classic LGMD with slowly progressive proximal muscle weakness (n = 8). Mild or nonspecific proximal weakness was reported in 12 cases. One case was explicitly described as not suggestive of LGMD [25], and another with pseudometabolic features, myalgia and fatigue [34]. Notably, one individual presented with ataxia in the absence of classical muscular dystrophy features [35].

Reported serum CK levels ranged from mildly elevated (~140 U/L) to over tenfold above the upper normal limit. Muscle imaging in one patient revealed selective involvement of posterior thigh and pelvic muscles [29]. WB data for calpain-3 were available for a limited subset of individuals (n = 8) and demonstrated variable findings. In one case, calpain-3 was absent [26], while another showed marked reduction [38]. The majority of individuals, however, showed normal calpain-3 expression [29,34,38]. One more detailed report described a reduction in the 94 kDa full-length band by 45.1%, with preservation of the 30 kDa autolytic fragment, a pattern indicative of retained proteolytic activity [28]. Consistent with this, functional assays of calpain-3 autolytic activity, performed in only two individuals, demonstrated preserved enzymatic function [38].

## 3. Discussion

Interpreting missense variants remains one of the most complex challenges in medical genetics. These single amino acid substitutions can variably affect protein structure, stability, interaction dynamics, and enzymatic activity and are often subtle and context-dependent, eluding current in silico predictions. The p.Asp753Asn variant in *CAPN3* is an illustrative example of this complexity. Although extensively reported in the literature data regarding its clinical, histopathological and biochemical features have been incomplete or inconsistently annotated, preventing a clear understanding of its pathogenic significance and mode of inheritance. The present study provides new and comprehensive insights into this variant through a multidisciplinary approach integrating clinical, histological, and structural analyses.

To address the complexity surrounding this variant, we compiled and integrated all available data on p.Asp753Asn carriers, combining findings from our cohort with reports from the literature. We observed that the frequent lack of detailed phenotypic annotation substantially hampers the establishment of a robust diagnostic framework for dominantly inherited calpainopathy. Moreover, the marked variability in both clinical manifestations and laboratory findings further underscores the challenges inherent in developing a reliable and standardized diagnostic algorithm for suspected LGMDD4. In our cohort, the clinical variability associated with this variant is evident: phenotypes ranged from asymptomatic hyperCKemia, to mild or moderate limb-girdle muscle weakness, to progressive weakness with contractures and respiratory involvement. Muscle biopsies showed a consistent but nonspecific myopathic pattern with low-grade structural alterations, while WB analyses demonstrated variable calpain-3 expression, normal in some, markedly reduced in others. Variability in calpain-3 protein levels may reflect not only the presence of the variant but also inter-individual differences and sample handling. Calpain-3 WB analyses have known limitations: for example, approximately 20% of individuals with pathogenic *CAPN3* variants may have normal protein levels despite functional impairment [14]. Tissue handling and preservation can also affect results, as partial thawing or exposure to moisture may promote artifactual autolysis, reducing detectable full-length protein [12,41]. Mechanistically, calpain-3 exists in dynamic oligomeric states, including dimers and higher-order assemblies such as trimers via the PEF domain [9,10]. Mutant and wild-type proteins may interact within these complexes, potentially affecting stability, turnover, or detection on WB. Notably, intrafamilial variability in calpain-3 expression has also been seen in dominant families, highlighting that protein levels can differ even among genetically related individuals [18]. Our observations align with previous reports of LGMDD4 [15,19,20] and reinforce the idea that diagnostic evaluation of suspected dominant calpainopathies should not rely solely on WB or CK levels. Individuals with single heterozygous *CAPN3* mutations may benefit from improved diagnostic evaluation through functional and imaging approaches reported in the literature. It has been demonstrated that standardized muscle MRI of the pelvis and lower limbs improves diagnostic accuracy and is especially useful when biochemical results are unclear. It usually reveals selective involvement of paraspinal, gluteal, hamstring, and medial gastrocnemius muscles [15,19,22]. Functional analyses such as those published by Vissing et al. [19], in which co-expression and activity assays demonstrated loss of mutant calpain-3 function along with dominant-negative reduction of wild-type protein, offer a useful example of assays that could be integrated into diagnostic workflows. Incorporating these additional tests can greatly boost diagnostic confidence when WB and CK results are insufficiently informative. Ultimately, comprehensive clinical, biochemical, and genetic correlation is essential for accurate diagnosis, particularly when evaluating single heterozygous variants with uncertain significance.

The p.Asp753Asn variant results in the substitution of a charged polar amino acid with a non-charged polar residue at position 753, which lies within the EF-hand domain, crucial for calpain-3 homodimerization [5,39]. Although previous structural modeling did not predict any major steric hindrance or disruption of substrate binding, it was previously hypothesized that the change in charge could modify the bond with other calpain-3 monomers [39]. Here we show that, although residue Asp753 is indeed present in the dimerization domain, it is located far away from the dimerization interface (Figure A1), thus excluding a putatively direct effect of p.Asp753Asn mutant in the dimerization process. Instead, our structural modeling provides novel insights into the potential molecular mechanism underlying the pathogenicity of p.Asp753Asn. In the wild-type protein, Asp753 forms a stabilizing salt bridge with Lys517, which contributes to maintaining inter-domain stability during activation. Substitution with asparagine removes the negative charge, likely disrupting this electrostatic interaction and weakening the structural coupling between domains. This disruption may alter the dynamic equilibrium between inactive and active conformations of calpain-3, impairing autolytic processing or substrate recognition. Such an effect is consistent with a dominant-negative mechanism, whereby the mutant protein interferes with the activity or stability of the wild-type enzyme. While this hypothesis requires experimental validation, it aligns with the emerging concept that certain *CAPN3* missense variants can exert dominant effects through altered dimerization or aberrant protease regulation. Although computational predictions and homology-based modeling cannot fully capture the complexity of calpain-3 dynamics, particularly in the absence of an experimentally resolved full-length structure, the consistency between our modeling data and the observed biochemical variability strengthens the plausibility of this structural interpretation. Future molecular dynamics simulations and in vitro functional assays will be necessary to confirm whether p.Asp753Asn perturbs calcium-dependent activation or dimer stability.

Recurrent detection of a given variant among unrelated affected individuals provides supportive evidence toward its pathogenic classification. Our literature review confirms that the p.Asp753Asn substitution is recurrently identified in unrelated individuals across diverse countries and diagnostic settings. Integration of our cohort data with published cases further demonstrates marked phenotypic variability, despite the frequent absence of detailed clinical information in many reports. A subset of patients presented atypical phenotypes, and at least two received an alternative diagnosis, underscoring how even mild or nonspecific manifestations, such as isolated CK elevation, may be overlooked when another disorder with more prominent clinical signs appears to better explain the presentation. Although LGMDD4 is typically a late-onset disease, a few pediatric-onset cases have been reported, including patients carrying c.643_663del21 or c.2440-1G>A variants and presenting with childhood-onset weakness and elevated CK [15]. This clinical overlap, combined with marked intrafamilial variability, suggests that genetic or environmental modifiers influence disease onset and progression and reinforces the notion of a spectrum of inheritance patterns including both dominant effects and contribution to recessive disease with a second pathogenic allele. Despite multiple independent reports, the precise molecular mechanism, particularly the hypothesized dominant-negative effect of p.Asp753Asn, remains unproven. Currently, no standardized or validated methods exist to directly demonstrate dominant-negative behavior in *CAPN3*-related disease. Functional studies capable of dissecting these effects at the cellular or molecular level are technically complex and rarely implemented in routine diagnostics. Consequently, many individuals carrying a single potentially pathogenic variant remain undiagnosed or misclassified.

The p.Asp753Asn variant exemplifies both the potential and the limitations of current genetic diagnostics. LGMDD4 represents a distinct and diagnostically challenging entity. Establishing pathogenicity requires integrated functional validation, family-based studies, and comprehensive phenotypic characterization. Future research should prioritize the development of standardized functional assays capable of detecting subtle alterations in calpain-3 activity and dimerization, alongside comprehensive phenotyping in larger, multicenter cohorts.

## 4. Materials and Methods

### 4.1. Subjects and Literature Review

The eight individuals carrying the c.2257G>A (p.Asp753Asn) variant included in the present study were drawn from a larger cohort of 59 patients with heterozygous *CAPN3* variants referred to five Italian neuromuscular centers (ClinicalTrials.gov NCT05956132). Inclusion criteria comprised patients heterozygous for a single *CAPN3* variant classified as pathogenic (class 5), likely pathogenic (class 4), or of uncertain significance (class 3), with no second *CAPN3* variant, as confirmed by MLPA, mRNA analysis, targeted neuromuscular gene panels, or whole-exome sequencing (WES). Demographic and pseudonymized clinical data were collected from medical records and included functional muscle assessments, serum CK levels, WB analyses, muscle biopsy findings, and muscle MRI.

For the literature review, we analyzed all published reports of the *CAPN3* c.2257G>A (p.Asp753Asn) variant, as documented ClinVar (accession no. VCV000281081.28). We identified 16 peer-reviewed publications originating from diverse geographic regions reporting a total of 31 cases carrying the p.Asp753Asn variant. These cases include both individuals with isolated heterozygosity and those with additional *CAPN3* variants in either compound heterozygous or complex allelic configurations.

### 4.2. Sequencing Analysis

Whole genomic DNA was extracted from peripheral venous blood samples using a standard procedure. Patients in this study were analyzed with a dedicated NGS panel for muscle diseases. We used SureSelect technology (Agilent, Santa Clara, CA, USA) and SureDesign software v. 8.0 (https://earray.chem.agilent.com/suredesign/ (accessed on 20 January 2025)) to design a multi-exon amplicon panel containing a total of 298 genes known to be associated with limb-girdle muscular dystrophies (LGMD), rhabdomyolysis, and metabolic and distal myopathies; the panel offered gene coverage greater than 99%. To analyze the data obtained from our study, we used a routine bioinformatics pipeline, QIAGEN Clinical Insight (QCI) Interpret software v. 9.2.1.20231012. Interpretation of the variants identified by this analysis is based on current knowledge and the ACMG classification [42]. The identified variants were validated by standard PCR-based capillary Sanger sequencing. The presence of a second pathogenic variant was excluded by MLPA according to standard procedures or by analysis of mRNA extracted from muscle tissue as previously described [43].

### 4.3. Calpain-3 Western Blot Analysis

Protein extracts were prepared from muscle biopsies of the patients as well as from healthy controls as described [44]. Briefly, comparable amounts of tissue from each sample were lysed in treatment buffer (0.125 mol/L Tris/HCl buffer, pH 6.4, 10% glycerol, 4% SDS, 4 mol/L urea), boiled for 2′ at 90 °C and centrifuged at 1300 rpm. An equal volume of 2× Laemmli Sample Buffer supplemented with 20% β-mercaptoethanol was added to each supernatant. Proteins were resolved on 4–15% TGX criterion midi gels in 1× TGS buffer for 2 h at 100 V. Dry transfer to a PDFV membrane was performed with the Trans-Blot Turbo Midi transfer pack for 20′ at 25 V. Gels were stained with SimplyBlue SafeStain. PVDF membranes were incubated with primary antibody (NCL-CALP-12A2, Leica Biosystems, Buccinasco, Italy) overnight at 4 °C. After incubation with HRP-conjugated anti-mouse secondary antibody (Life Technologies # 31450, Segrate, Italy) chemiluminescent detection was performed with Clarity™ Western ECL Substrate (BioRad # 1705060, Segrate, Italy) and acquisition was performed with the iBright Imaging System CL1000 (Thermo Fisher Scientific by Life Technologies, Segrate, Italy).

### 4.4. Structural Modeling

To be consistent with previous works [20,45] and to allow comparison, the calpain 3 structure was generated by using protein structure homology modelling using SWISSMODEL workspace [46] and visualized by Chimera 1.19 software (UCSF) [47]. In order to assess the putative role of the Asp753Asn mutation, two models were generated: Ca^2+^ bound state and Ca^2+^ unbound state. The templates chosen for both cases were the M-calpain in Ca^2+^ bound and unbound states (PDB 3BOW and 1KFU, respectively), sharing a sequence identity of 52% with calpain 3. The movie was created by using the ‘Morph conformation’ program within the Chimera software [48]. To gain deeper insights into the multimer formation and putative effect of the mutation in the dimerization process, we have modelled the full-length dimer using Alphafold 2 (https://alphafoldserver.com/ (accessed on 25 February 2025)).

## 5. Conclusions

Dominant calpainopathies, particularly those linked to single missense variants such as p.Asp753Asn, remain a diagnostically challenging entity. Their pathogenicity cannot rely solely on variant type or zygosity but must be interpreted through functional studies, segregation analyses, and detailed phenotypic evaluation. Until integrated datasets become widely available, caution in the interpretation is warranted. Developing robust functional assays and standardized diagnostic algorithms will facilitate accurate genetic counseling and enable patient-specific rehabilitation strategies aimed at preserving muscle strength and function. Beyond diagnostics, these efforts will improve understanding of how CAPN3 variants perturb calpain-3 activity, stratify patients for future clinical trials, and ultimately guide the development of targeted therapies.

## Figures and Tables

**Table 1:** Clinical and histopathological features of patients carrying the *CAPN3* c.2257G>A (p.Asp753Asn) variant. Demographic, clinical, and pathological data are presented for eight individuals in our cohort. LL = lower limbs; UL = upper limbs; p = proximal; d = distal; R = right; L = left, and NP = not performed.

Patient	Sex	Onset (Years)	CK	First Symptoms	Neurological Evaluation	MRI/CT Scan	Biopsy	WB Calpain-3
P1	M	46	4×	Asymptomatic	Unremarkable	Unremarkable	Myopathic	NP
P2	F	49	Normal	Waddling gait	Progressive pLL > pUL weakness	NP	Myopathic	Normal
P3	F	56	Normal	pLL > pUL	Progressive mild pLL weakness and bent spine	Fibro-fatty replacement dorsal and lumbar paraspinal, gluteal and biceps femoris	Myopathic	Reduced
P4	M	50	4×	Stepping and waddling gait	Progressive dLL > pLL + dUL	Unremarkable	Myopathic	Reduced
P5	M	/	2×–4×	Myalgia	Unremarkable	Unremarkable	NP	NP
P6	M	6	Normal	Myalgia and exercise intolerance	Unremarkable	NP	NP	NP
P7	M	40	5×	Difficulty in walking and frequent falls	Severe pLL > dLL + pUL + axial weakness; dysphagia and respiratory involvement	Diffuse fibro-fatty replacement	Myopathic	Normal
P8	M	60	5×	Myalgia and fatigue	Mild pLL weakness, bilateral pes cavus (R > L)	Diffuse fibro-fatty replacement postero-lateral compartment tight and calf	Myopathic	Normal

## Data Availability

The original contributions presented in this study are included in the article/Supplementary Materials. Further inquiries can be directed to the corresponding author.

## Supplementary Materials

The following supporting information can be downloaded at: https://www.mdpi.com/article/10.3390/ijms262311384/s1.

## Abbreviations

The following abbreviations are used in this manuscript:

LGMD	Limb-Girdle muscular dystrophy
WB	Western blot
WES	Whole exome sequencing
CK	Creatine kinase

## Appendix A

**Table A1:** Reported cases of the *CAPN3* p.Asp753Asn variant in the literature. Summary of reported cases across 16 studies published between 2004 and 2024. Clinical phenotypes, CK levels, muscle biopsy findings, and Western blot results are reported as originally stated by authors. No. PZ indicates the number of individuals with the p.Asp753Asn substitution reported in the referenced study. No. in Study refers to the specific patient numbering adopted within the original publication. CK: creatine kinase; WB: Western Blot; LGMD: limb-girdle muscular dystrophy; NGS: next-generation sequencing; WES: whole-exome sequencing; MLPA: multiplex ligation-dependent probe amplification; SSCP: single-strand conformation polymorphism; ARMS-PCR: amplification-refractory mutation system PCR; HT-DHPLC: high-throughput denaturing high-performance liquid chromatography. Empty fields “/” indicate unavailable data.

Ref	No. PZ	No. in Study	Sex	Family History	Onset (Years)	Phenotype	Clinical Features	Severity	CK	WB Calpain-3	Additional Mutations	Genetic Analysis
[26,38,40]	4	52	F	N	3	LGMD	LGMD	Moderate	/	Absent	No	SSCP, ARMS-PCR, SANGER HT-DHPLC
		71	/	/	/	/	/	/	/	Normal	c.1355-6G4T	
		48	/	/	/	/	/	/	/	Reduced 5%	c.2242C4T (p.R748X)	
		88	/	/	/	LGMD	HyperCKmia	Mild	High	Normal	No	
[25]	1	/	/	/	40	Not suggestive of LGMD	/	Benign evolution	/	/	No	SSCP, Sanger
[27]	1	18	F	N	25	Mild proximal weakness	Difficulty climbing stairs	Moderate	140 U/L	/	No	Sanger
[28]	1	XXXV	F	N	37	LGMD	/	Slow progression	10–13×	94 kDa Reduced 45.1%, 30 kDa Normal	No	Sanger
[29]	2	19	/	N	40	LGMD	Pelvic girdle onset, Mild facial involvement	Mild, slow/moderate progression	2×	Normal	No	Sanger
		20	/	N	55	LGMD	Pelvic girdle onset	Mild, slow/moderate progression	1.5×	Normal	No	
[17]	2	C21	/	/	/	/	/	/	/	/	No	NGS panel
		C36	/	/	/	/	/	/	/	/	No	
[30]	1	P19	M	/	39	Asymptomatic	HyperCKmia	Asymptomatic	2000 U/L	/	c.1395-1397del (p.Leu465_Glu466del); c.1453A>G (p.Met485Val)	NGS panel
[33]	2	/	/	/	/	/	/	/	/	/	N/A	WES
[31]	10	/	/	/	/	Proximal weakness	/	/	/	/	N/A	NGS panel
[32]	1	P8	M	/	/	/	/	/	Normal	/	No	NGS panel, Sanger, MLPA
[34]	1	A13	F	N	30	Pseudometabolic	Paternal inheritance, Myalgia, fatigability	Mild	6×	Normal	g.42350479A>G; g.42354194C>T	WES
[35]	3	29	M	/	21	ataxia	/	/	/	/	No, carrying other causative variants in other genes	Custom Target Capture NGS panel
		32	M	/	2	/	Difficulty climbing stairs	/	High	/	No, carrying causative variants in other genes	
		35	M	/	1	/	Hypotonia	/	400 U/L	/	No	
[36]	1	U12	M	/	3	Asymptomatic	HyperCKmia	Asymptomatic	High	/	Not found	NGS panel, Sanger
[39]	1	4	F	/	40	LGMD	Scapular winging, waddling gait, severe upper and lower girlde weakness	Severe	1169 UI/L	/	c.967G>T (p.Glu323*), c.1401_1403delGGA (p.Glu467del)	Sanger
