# Supplementary material for: Recurrent CAPN3 p.Asp753Asn Variant Supports a Potential Dominant Calpainopathy with Variable Clinical Expressivity
Source: Int J Mol Sci. 2025 Nov 25;26(23):11384. doi: 10.3390/ijms262311384 (PMC12692566; doi:10.3390/ijms262311384)
Supplement: Supplementary file 1 [file ijms-26-11384-s001.zip › Caption supplementary files.pdf]

Supplementary Video S1. Calpain-3 Inter-Domain Conformational Changes. Simulation of the conformational change of the inter-domain organization from the calcium-free structure to the calcium-bound structure of calpain 3 created by using the 'Morph conformation' program within the Chimera software, which creates a trajectory that morphs between two structures, i.e., the models of the Ca^{2+} unbound and bound states, as the starting and final configurations, respectively. It can be clearly observed that the domains shift relative to each other to produce the more compact, active enzyme.
